# How to Value Orphan Drugs? A Review of European Value Assessment Frameworks

**DOI:** 10.3389/fphar.2021.631527

**Published:** 2021-05-12

**Authors:** Alessandra Blonda, Yvonne Denier, Isabelle Huys, Steven Simoens

**Affiliations:** ^1^ Department of Pharmaceutical and Pharmacological Sciences, KU Leuven, Leuven, Belgium; ^2^ Department of Public Health and Primary Care, KU Leuven, Leuven, Belgium

**Keywords:** orphan drug, rare disease, value assessment framework, multi-criteria decision analysis (MCDA), decision-making, economic evaluation, health technology assessment (HTA), fairness

## Abstract

**Background:** Decision-makers have implemented a variety of value assessment frameworks (VAFs) for orphan drugs in European jurisdictions, which has contributed to variations in access for rare disease patients. This review provides an overview of the strengths and limitations of VAFs for the reimbursement of orphan drugs in Europe, and may serve as a guide for decision-makers.

**Methods:** A narrative literature review was conducted using the databases Pubmed, Scopus and Web of Science. Only publications in English were included. Publications known to the authors were added, as well as conference or research papers, or information published on the website of reimbursement and health technology assessment (HTA) agencies. Additionally, publications were included through snowballing or focused searches.

**Results:** Although a VAF that applies a standard economic evaluation treats both orphan drugs and non-orphan drugs equally, its focus on cost-effectiveness discards the impact of disease rarity on data uncertainty, which influences an accurate estimation of an orphan drug’s health benefit in terms of quality-adjusted life-years (QALYs). A VAF that weighs QALYs or applies a variable incremental cost-effectiveness (ICER) threshold, allows the inclusion of value factors beyond the QALY, although their methodologies are flawed. Multi-criteria decision analysis (MCDA) incorporates a flexible set of value factors and involves multiple stakeholders’ perspectives. Nevertheless, its successful implementation relies on decision-makers’ openness toward transparency and a pragmatic approach, while allowing the flexibility for continuous improvement.

**Conclusion:** The frameworks listed above each have multiple strengths and weaknesses. We advocate that decision-makers apply the concept of accountability for reasonableness (A4R) to justify their choice for a specific VAF for orphan drugs and to strive for maximum transparency concerning the decision-making process. Also, in order to manage uncertainty and feasibility of funding, decision-makers may consider using managed-entry agreements rather than implementing a separate VAF for orphan drugs.

## Introduction

Rare diseases are a group of diverse diseases, each characterized with low prevalence: occurring in less than one in 2,000 people in Europe (European Medicines Agency 2007). They are defined as life-threatening or chronically debilitating, and are mostly caused by a genetic predisposition (Nguengang Wakap et al., 2020). For a long time the needs of rare disease patients were neglected by pharmaceutical companies negating investment in treatment for these, as they anticipated insufficient return by such a small group of patients (European Commission n.d, 2000; Griggs et al., 2009) as well as impractical requirements from regulatory authorities. Nevertheless, rare diseases pose a high burden on patients, as they often need lifelong treatment and care. The severity of many of these diseases limits the patient’s social, educational and professional lives. As a result, they often have lower wages while being confronted with relatively high additional costs, compared to patients suffering from a non-rare disease. Moreover, they highly depend on their caregivers, often family members, to perform daily tasks. As a result, a rare disease also limits the caregivers’ professional activity, as they spend significant time on care-related tasks (EURORDIS, 2017).

Towards the end of the 20th century, patients with various rare diseases organized themselves based on their experiences with similar issues: the feeling of being invisible to public health systems (resulting in) a large unmet medical need (due to a lack of therapeutic alternatives) and a joint feeling of being treated unfairly compared to non-rare disease patients (Huyard, 2009). Their initiative had a major impact on regulatory policy in both the United States and Europe. In 2000, the EU adopted legislation in order to provide incentives for manufacturers investing in Orphan Medicinal Products (OMPs) (Huyard, 2009). The “Orphan Medicinal Product Regulation” defines OMPs as products for the “diagnosis, prevention or treatment of life-threatening or very serious conditions that affect no more than 5 in 10,000 people in the European Union”.^1^ It provides fee waivers for regulatory procedures, protocol assistance and a 10-years market exclusivity after authorization by the EMA (European Medicines Agency, 2007). These incentives led to an increase in OMP development, with currently 1,705 products designated as OMPs in the EU, of which 191 are currently authorized (European Commission n.d, 2016). Yet despite all efforts, patient access to OMPs remains an issue. Studies show significant variations in OMP access across countries (Szegedi et al., 2018; Picavet et al., 2012a; Annemans, et al., 2017; Pejcic et al., 2018; Zamora et al., 2019). For instance, in 2019, the Netherlands reimbursed all but three of the 164 registered EU OMPs, compared to 70 reimbursed OMPs in Romania (Czech et al., 2020). These variations may be due to the way in which these drugs are appraised, as decision-makers often rely on a country specific health technology assessment (HTA). In an HTA, a drug’s performance is assessed by several criteria, which mainly focus around the drug’s safety, efficacy and economic consequences of its reimbursement (such as cost-effectiveness and budget impact). Although these may be considered to be the traditional HTA criteria, others criteria may apply as well. However, as often the acquisition costs of OMPs are high and their (cost-) effectiveness (at least at the time of submission) is uncertain, decision-makers struggle to reimburse them through their standard assessment and subsequent appraisal processes (Drummond et al., 2007). Furthermore, HTA processes are not harmonized across countries, which may lead to different reimbursement decisions of OMPs (Stawowczyk et al., 2019; Zamora et al., 2019; Czech et al., 2020).

In order to account for the specific characteristics of OMPs and of rare diseases, decision-makers are increasingly adapting their reimbursement processes (Nicod et al., 2019). This has resulted in a variety of different approaches toward OMP assessment, which we will further refer to as value assessment frameworks (VAFs). Through these adapted VAFs, decision-makers attempt to balance standard efficiency criteria such as cost-effectiveness with additional, not traditionally used criteria, such as severity and unmet need. Efforts are being made by EMA and EUnetHTA in order to streamline the process of market authorization and reimbursement across European jurisdictions (European Medicines Agency and EUnetHTA, 2020). In the meantime, however, these different approaches toward OMP appraisal risk to further contribute to the unequal access of OMPs for patients between jurisdictions. Moreover, they create an unpredictable environment for manufacturers, who may invest a significant amount into the development of OMPs, while being unable to predict whether their investment will ultimately lead to reimbursement. Yet, fostering innovation is absolutely necessary, given the fact that there is still no authorized treatment for most rare diseases. The crux of the matter is that, while decision-makers ideally appraise the OMP according to their formal VAF and its evaluation criteria, some have modified these VAFs in order to take other appraisal criteria into consideration. These modifications may complicate a proper comparison of the VAFs between jurisdictions. Yet in general, VAFs that have been applied in the context of OMPs are either those with or without a standard economic evaluation, frameworks that attach weights to quality-adjusted life years (QALYs) or allow a higher threshold of the incremental cost-effectiveness ratio (ICER), or rather conceptual frameworks such as multi-criteria decision-analysis (MCDA), in which drugs are appraised according to an explicit yet flexible set of criteria, or any combination thereof. Previously, researchers in the field of health policy have mainly focused on the appraisal criteria in the context of OMPs (Zelei et al., 2016; Bourke et al., 2018; Picavet, et al., 2014a; Szegedi et al., 2018; Nicod et al., 2017), arguments in favor or against a special reimbursement status for OMPs (Simoens et al., 2012; Hughes et al., 2005; Picavet, et al., 2012b), proposals concerning existing or conceptual VAFs tailored to the needs of OMPs (Sussex et al., 2013b; Wagner et al., 2016; Annemans et al., 2017; Schey et al., 2017), or discuss ethical, social, or other features of specific VAFs for OMPs (Drummond and Towse 2014; Simoens 2014; Schlander et al., 2016; Towse and Garau 2018). Most recently, a study was published by Nicod et al. mapping the different VAFs that jurisdictions have implemented (Nicod et al., 2020). As of yet, however, no publication exists that provides an overview of the strengths and weaknesses that are associated with each of these VAFs. Nevertheless, such an overview could be highly valuable to decision-makers who wish to adapt or reflect on their current VAF. Also, as the marketing authorization and reimbursement processes are further aligned, questions may arise about when and how clinical and economic data may be considered (European Medicines Agency and EUnetHTA, 2020). In order to fill this gap, our review aims to identify and discuss the arguments in favor or against the various VAFs that can be applied to OMPs, by means of a narrative literature review. Subsequently, we have illustrated these arguments by examples of OMP VAFs implemented in European jurisdictions.

## Methods

### Search Strategy

The search strategy focused around two co-occurring concepts: “orphan drug” and “value assessment framework”. Through an iterative process we identified a set of synonyms for each term. For the concept “orphan drug”, we included the synonym “orphan medicinal product”. For the concept “appraisal”, synonyms were considered such as “value assessment”, “framework”, “appraisal” “cost-effectiveness”, “health-technology assessment”, “economic evaluation”, “economic”, and “MCDA”. Accordingly, we performed the search in Pubmed (MEDLINE and non-MEDLINE), Web of Science (WoS) and Scopus, including all types of study design (opinion pieces, commentary, editorial, systematic, narrative, or scoping reviews, etc.). Language of the studies was limited to English. Since the EU Orphan Drug Directive was implemented in 2000, we included publications between the 1st of January 2000 and the August 22, 2020.

### Article Selection

To date, only a limited amount of publications discusses the strengths and weaknesses of VAFs in the context of OMPs. Most arguments are mentioned in the body text of articles discussing OMPs or VAFs in general, or embedded in ethical discussions thereof. For this reason, we have adopted broader inclusion criteria during the (record) screening phase of our literature search, including articles that discuss the assessment or appraisal process for the reimbursement of OMPs, societal preferences toward OMP or their economic evaluation. However, we included arguments in our study when they were relevant to the appraisal of OMPs. Additionally, we included studies previously known to the authors and those that were identified through snowballing. Additional focused Google searches were performed to include gray literature, such as news articles or publications of reimbursement and HTA-agencies, that mention strengths or barriers of VAFs that may apply for OMPs. These searches included combinations of keywords as for instance “weakness” + “variable ICER” + “the Netherlands” or for instance “strength” + “weighted QALY” + “Norway”.

### Concepts and Categorization

First of all, in the context of this manuscript, we will refer to the term “jurisdiction” as the territory that falls under the responsibility of an HTA body. Also, in the following sections, we will refer to the term “value assessment framework” as the way in which all appraisal criteria are brought together, and the performance of the medicinal product against these criteria is discussed, in order to decide on the product’s reimbursement. In the context of OMPs, a decision-making body may choose to change or include other criteria or approaches toward assessment or appraisal of these criteria, as a means to tailor their standard VAF to the needs of OMPs. For example, the applicant may be allowed to submit data from observational studies rather than from a clinical trial, or they may allow a higher ICER according to the disease’s severity. We consider the combination of all of these adaptations in a given jurisdiction to be the VAF for OMPs (or ultra-OMPs). In this context, we have identified and categorized what we believe to be the main VAFs for OMPs. For each VAF, we have provided some examples of jurisdictions where these VAFs are implemented. These examples were chosen depending on the available data on VAFs across geographical Europe. These, together with a concise definition of each of the VAFs, will allow a clear illustration of the VAF’s strengths and weaknesses.

## Results

### Search Results

**FIGURE 1:**
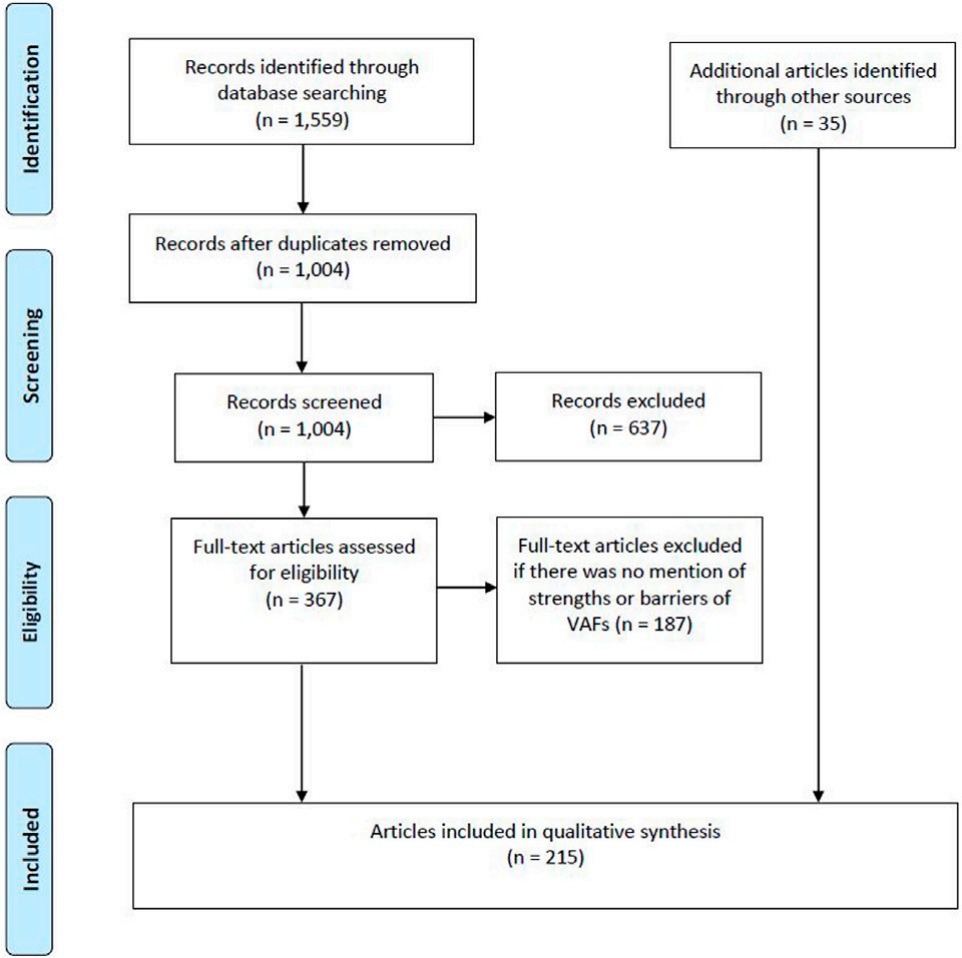
PRISMA flow diagram of search strategy results.

Our literature search yielded 1,559 articles. We excluded articles that focused solely on the regulatory process for OMP designation or authorization, on the availability of OMPs, on price comparisons of OMPs, and on managed entry agreements and/or risk sharing schemes. Afterward, a full-text screening was performed of which we excluded publications that did not mention any strengths or barriers of VAFs for OMPs. Additional publications were added either through snowballing, focused searches or because they were known by the authors. In total, 215 publications were selected to be included in the study (see Figure 1).

### Defining the Value Assessment Frameworks for Orphan Drugs

In Table 1 we have provided a description for each of the six VAFs that we have categorized in the context of OMPs. The first four VAFs, either without economic evaluation, with a standard economic evaluation, using a modified ICER threshold or QALY weights (see *Frameworks That Do not Apply Economic Evaluation, Applying a Standard Economic Evaluation, Modifying the ICER Threshold, Attaching Weights to QALYs*), relate primarily to the VAF’s approach toward the assessment of specific criteria such as cost-effectiveness or disease severity, whereas the last two, the concept of MCDA and a separate VAF for ultra-OMPs (see *Multi-Criteria Decision Analysis (MCDA), A Separate Framework for Ultra-OMPs*) relate particularly to how the criteria and multiple approaches are assembled within the VAF. These 6 categories are not all mutually exclusive, meaning that a jurisdiction’s VAF may fall under multiple categories (see *Combination of Value Assessment Frameworks*).

**TABLE 1:** An explanation of the different value assessment frameworks in the context of (ultra-)OMPs and their implementation in jurisdictions across geographical Europe.

Value assessment framework	No economic evaluation	Standard economic evaluation	Variable ICER threshold	Weighted QALYs
Definition	Evaluates an intervention by considering evaluation criteria other than cost-effectiveness, such as efficacy and effectiveness, safety, feasibility or added therapeutic value compared to the standard of care (SoC)	Compares both an intervention’s cost and effectiveness against the current SoC. Outcome is the incremental-cost-effectiveness ratio (ICER, i.e. the extra cost we have to pay in order to gain a unit of health benefit over the existing alternative), compared to a benchmark (the ICER threshold) or to the ICERs of other interventions funded by the health budget (Simoens 2009)	Compares an intervention to the SoC, hereby allowing the ICER threshold to change according to predetermined appraisal criteria or societal preferences, such as prevalence, severity of illness, fair innings (higher priority to treatments for patients with a severely life-shortening disease) or a disease’s social value (Williams 1997; Towse and Garau 2018; Drummond et al., 2009). For each preference, separate priority classes can be created for which a different threshold applies. Ideally, this evaluation process is separated into two independent, yet simultaneous assessments: One to define the priority class and another to perform the economic evaluation (Nord et al., 2009)	Compares an intervention to the SoC, hereby increasing the weight of a unit of health benefit (the quality-adjusted life year, QALY) according to predetermined appraisal criteria such as disease severity or unmet medical need. A common approach is to capture societal preferences, transform them into weights and multiply the number of QALYs gained with the relevant equity weight. The outcome is a new ICER that can then be compared with the standard cost-effectiveness threshold (Wailoo et al., 2009). For other approaches to weighting QALYs, we refer to the article by (Nord et al., 2009)
Examples	- Belgium: Cost-effectiveness of orphan medicinal products (OMPs) and non-OMPs not considered a decision-making factor for their reimbursement (Nicod and Whittal, 2020)	- Austria, Bulgaria, Scotland, Ireland (Nicod and Whittal, 2020), Latvia (Malinowski et al., 2019; Nicod and Whittal, 2020), Liechtenstein, Malta, Poland, Portugal	- Slovakia: Variable ICER threshold for non-OMPs and OMPs (Nicod and Whittal, 2020)	- England and Wales: QALY weights apply if (i) the ICER exceeds £100,000/QALY and (ii) there is strong evidence that the treatment offers significant QALY gains compared to its competitor (see Table 3 for QALY weights) (National Institute for Health and Care Excellence (NICE) 2017)
	- France: Cost-effectiveness analysis (CEA) considered when the budget impact (BI) exceeds 20 million (Nicod and Whittal, 2020)		- Sweden: Variable ICER threshold applied for all interventions (Nicod and Whittal, 2020)	
	- Germany: Cost-benefit analysis (CBA) performed for an OMP only when its estimated annual turnover exceeds €50 million (European commission (EC) 2016; Knight and Pearson, 2019)	- Romania: Adopted for all interventions	- The Netherlands: Variable ICER threshold for all interventions according to disease severity, with an ICER threshold of 20.000, 50.000 or 80.000 when severity falls between 0.1 and 0.4, 0.41, and 0.71, and 0.7 and 1 respectively (Stolk et al. 2004; van de Wetering et al., 2013; Wetering et al., 2015; Kanters, 2016)	
	- The Netherlands: Cost-effectiveness of both non-OMPs and OMPs is considered only when the budget impact is high (>50 million), or when there is a high price per patient per year combined with a budget that exceeds 10 million (Nicod and Whittal, 2020)	- Lithuania: Adopted for non-OMPs and OMPs, not for ultra-OMPs (Nicod and Whittal, 2020)	- Scotland: a Higher threshold may be accepted for OMPs and ultra-OMPs (Nicod and Whittal, 2020)	
	- Slovakia and Lithuania: Cost-effectiveness not requested for ultra-OMPs (Malinowski et al., 2019; Nicod and Whittal, 2020)		- Norway: a Higher ICER threshold may be accepted for ultra-OMPs (Wiss 2017)	

Value assessment framework	Multi-criteria decision analysis (MCDA)	Separate VAF
Definition	A matrix in which multiple elements of value, such as cost-effectiveness, disease severity and unmet need, can be included to generate a composite score for an intervention. A multidisciplinary team first identifies all relevant criteria of value (appraisal criteria). Criteria are then prioritized, by attaching weights according to relevance for the treatment under evaluation. Afterwards, the performance of the intervention against each criterion is scored. The final result is an overall score which can be used to identify the most preferred treatment for funding or make a list in which treatments are ranked (Thokala and Duenas, 2012; Belton and Steward, 2002; Dodgson et al., 2009)	Appraises an intervention through a value assessment framework (VAF) that is different from the standard VAF
Examples	- Slovakia: “MCDA-like approach” for non-OMPs and OMPs, as they add weights to appraisal criteria. An MCDA-score is used to vary the ICER threshold	- France, Lithuania and Slovakia: Ultra-OMPs do not pass through the standard VAF, yet it’s not clear whether other appraisal criteria are considered (Nicod and Whittal, 2020)
	- Romania: “MCDA-like approach” that adds weights to appraisal criteria. No formal health technology assessment (HTA) process in place, but uses a “score cards” HTA method for both OMPs and non-OMPs. Through the VAF, each drug receives points according to a specific set of criteria (including cost-effectiveness) (Radu et al., 2016; Malinowski et al., 2019). Bonus points are granted for OMPs, which are approved only after a special therapeutic protocol is set-up (Radu et al., 2016; Malinowski et al., 2019; Nicod and Whittal, 2020)	- England and Wales: Separate VAF, highly specialized technologies (HST) appraisal process, implemented by the National Institute for Health and Care Excellence (NICE). The VAF applies for (ultra-)OMPs that meet a certain set of requirements such as “the target patient group for the technology in its licensed indication is so small that treatment will usually be concentrated in very few centers in the NHS” (National Institute for Health and Care Excellence (NICE) 2017). The pathway considers additional appraisal criteria such as the nature of the condition (including the impact of the disease on caregivers’ quality of life), cost/QALY, and the impact of the technology beyond direct health benefits (National Institute for Health and Care Excellence (NICE) 2017)
	- Lombardy in Italy: The *Valutazione delle Tecnologie Sanitarie* (VTS) framework adopted the EUnetHTA^®^ core model to guide the decision-making process Radaelli et al. (2014). Consists of a prioritization, assessment and appraisal step. Appraisal step is guided by an EVIDEM-like approach toward MCDA. Compared to EVIDEM changes were made to the criteria in order to avoid mentioned shortcomings such as the mutual independence and overlap between criteria. Incorporates an opportunity cost approach, requiring applicants to highlight which interventions will be substituted (and thus, may become candidates for disinvestment) Cleemput et al. (2011), Radaelli et al. (2014)	- Scotland: Separate VAF for OMPs and ultra-OMPs (and end-of-life medicines), implemented by the scottish medicines consortium (SMC) (Scottish Medicines Consortium (SMC) n.d.). Similar appraisal criteria to those of the HST process apply, yet SMC applies a more qualitative approach, and may thus accept a higher threshold compared to OMPs and non-OMPs Nicod and Whittal (2020)

HTA, health technology assessment; ICER, incremental cost-effectiveness ratio; MCDA, multi-criteria decision analysis; NICE, National Institute for Health and Care Excellence; OMP, orphan medicinal product; QALY, quality adjusted life-year; SMC, Scottish Medicines Consortium; SoC, standard of care; VAF, value assessment framework.

### Strengths and Weaknesses of Value Assessment Frameworks for Orphan Drugs

Table 2 presents, for each VAF, a summary of the strengths and weaknesses in the context of OMPs. They are explained in detail in the following sections.

**TABLE 2:** A summary of value assessment frameworks, their strengths and weaknesses, in the context of (ultra-)OMPs.

Value assessment framework	No economic evaluation	Standard economic evaluation	Variable ICER threshold	Weighted QALYs	Multi-criteria decision analysis	Separate VAF
Strengths	- Allows flexibility to reimburse (ultra-)OMPs regardless of its cost-effectiveness	- Subjects all drugs to the same cost-effectiveness standards	- Increases chance for reimbursement	- Increases chance for reimbursement	- Flexibility to in-and exclude criteria	- Might meet some of the shortcomings of other VAFs
		- May motivate manufacturers to improve methods to reduce data uncertainty	- Implications of considering non-traditional criteria such as disease severity and unmet need are made explicit		- Supports making trade-offs between competing values through criteria weighting	
		- Includes different perspectives (for instance, health care payer or societal perspective)			- Considers all criteria consistently in an explicit manner	
					- Increases transparency as key decision-making arguments become traceable	
					- Allows interpretation of data by multiple stakeholders	
					- Provides legitimacy of a final decision	
					- Manages data uncertainty accordingly	
					- In time: may increase consistency between appraisals, provide insight into (country specific) societal preferences, direct investments toward criteria with higher value	
Weaknesses	- Reimbursing cost-ineffective (ultra-)OMPs risks decreasing population health	- A universal and constant ICER threshold does not exist	- Increases inequality when methodology is flawed	- Increases inequality when methodology is flawed	- No consistency between frameworks	- Lack of consensus on the importance of rarity in prioritizing funding
		- Less likely for OMPs to meet common ICER thresholds	- Societal preference studies, which determine criteria, contain flaws	- Societal preference studies, which determine criteria, contain flaws	- Issues with criteria validity and overlap	- Requirements to enter separate pathway are often vague
		- Methods to value the QALY are flawed, do not capture full value	- Increasing ICER threshold may demotivate cost-effective OMP development	- Increases complexity when multiple appraisal criteria are considered simultaneously	- No benchmark to compare MCDA scores with	- Time to reach final decision may be too long
		- QALY value depends on the individual’s characteristics			- May decrease importance of cost-effectiveness and feasibility criteria	
		- Creates unequal access to treatment			- Reluctancy towards more transparency	

#### Frameworks That do not Apply Economic Evaluation

Faced with increasingly constrained healthcare budgets, decision-makers in most European jurisdictions consider an OMPs cost-effectiveness when deciding on reimbursement. However, some countries have not incorporated cost-effectiveness into their standard assessment process or may exempt either OMPs, ultra-OMPs or both from their standard approach (see Table 1).

When decision-makers choose not to subject OMPs to an economic evaluation, it may allow them to reimburse (ultra-)OMPs that have unfavorable cost-effectiveness. One of the reasons behind this argument is the fact that they are often regarded as being highly priced while their effectiveness is uncertain at the time of submission (Schuller et al., 2015; Drummond et al., 2007). We refer to Box 1, where we have further explored the arguments behind this statement. At the same time, most societies adopt a utilitarian perspective toward healthcare, which means that decision-makers aim to maximize health benefits within a limited budget. If a payer would grant reimbursement to an OMP that is not effective, this would imply that these funds cannot be spent on other treatments that are cost-effective (Picavet et al., 2014b). Such VAFs risk decreasing population health if (cost-ineffective) OMPs are reimbursed (Ollendorf et al., 2018).

Box 1 **Clinical uncertainty and the black box of OMP pricing.**

Compared to non-OMPs, several authors consider an OMPs effectiveness to be more uncertain, as clinical data is often limited at time of submission. This is due to the rarity of the disease, affecting very small yet heterogeneous patient groups, thereby creating a lack of knowledge on the natural history of the disease, a lack of clinical expertize and hence, a great difficulty in establishing appropriate (surrogate) clinical trial endpoints. Also, due to low patient numbers and difficulty recruiting patients, clinical trials for OMPs are generally smaller. Furthermore, due to the high unmet need, they are less likely to include a comparator/placebo arm. On top they run shorter, since the disease’s severe nature and unmet need increase the urgency to market the OMP (Nestler-Parr et al., 2018; Hughes et al., 2005; Schlander et al., 2016; McCabe et al., 2006; Lagakos 2003; McCabe et al., 2007; Augustine et al., 2013; Hughes-Wilson et al., 2012; Pearson et al., 2018). On the other hand, the pricing of OMPs is, to date, perceived to be a black box (Picavet et al., 2013). OMP prices are set relatively high when compared to non-OMPs, with manufacturers claiming they need to recoup high acquisition costs from a limited number of patients. By increasing the OMP’s unit price, they may attempt to decrease their financial risk (Drummond et al., 2007; McCabe et al., 2010; Schlander et al., 2016). The high unit price may also include other substantial cost-drivers, such as expensive post-marketing surveillance programs (Simoens 2011; Schlander et al., 2016) and extra costs linked to the adaptation to the different national pricing and reimbursement procedures (Boon and Moors, 2008). Finally, the monopolistic position of many OMPs may also contribute to higher prices when, among other reasons, a high unmet need creates a higher willingness to pay (Boon and Moors, 2008; Simoens 2011).

#### Applying a Standard Economic Evaluation

To determine whether an intervention provides value for money, decision-makers can rely on an economic evaluation (see Table 1). A framework that utilizes a standard economic evaluation generally treats both OMPs and non-OMPs in the same way. However, there is still much debate as to whether OMPs deserve a special treatment over non-OMPs in reimbursement procedures, with authors arguing in favor (Drummond et al., 2007; Simoens et al., 2012), or against (McCabe et al., 2005).

From the available literature, we have deducted three main strengths of using standard economic evaluation for the assessment of OMPs. First of all, a standard economic evaluation holds all drugs to the same standards (McCabe et al., 2006), and hereby guarantees an equal treatment of OMPs vs. non-OMPs. Given the fact that it is currently unclear whether society prefers to fund treatment by OMPs (see Box 2) (Richardson and Schlander 2019), by subjecting both to the same cost-effectiveness standards, payers make sure that the “*anonymous many are not harmed to benefit the identifiable few*”(McCabe et al., 2010).

Box 2 **Does society wish to prioritize treatment for a rare disease?**

Critics of standard economic evaluation often argue that the approach toward OMPs should differ from those toward non-OMPs because of the rarity of the disease an OMP treats. Rarity is the only characteristic that separates an OMP from a non-OMP, and is as such captured in OMP legislation (Regulation (EC) No 141/2000). Nevertheless, several social preference studies have indicated that society does not wish to prioritize funding of OMPs over non-OMPs based purely on the rarity of the diseases they treat (Desser et al., 2010; National Institute for Clinical Excellence (NICE), 2004; McCabe et al., 2010; Ryan et al., 2001; Bourke et al., 2018). Then in 2019, Richardson and Schlander found that the outcome of societal preference studies is influenced by the way the questions are framed. They concluded that in some cases citizens may prioritize funding for cost-ineffective OMPs, when there is only a small impact for each citizen baring the costs (Richardson and Schlander 2019). They state that, when study questions are developed from a utilitarian rather than a rights-based perspective (which aims to maximize equity by allocating resources fairly), the attributes under study (which define the social preferences) are framed. For instance, questionnaires usually mention costs from the perspective of the interviewee as a patient, rather than from the interviewee as a citizen and taxpayer. An interviewee acting as a citizen tends to be more sharing than an individual patient, indicating that the chosen perspective influences the interviewees’ behavior (Richardson and Schlander 2019). This implies that researchers should be careful when developing questionnaires that aim to define any preference toward disease rarity and should validate them.

Also, requiring OMPs to adhere to standard cost-effectiveness thresholds may motivate manufacturers to improve methods for the collection of robust study data and, subsequently, reduce the uncertainty regarding the clinical effectiveness of OMPs (Berdud et al., 2020). Another strength of a standard economic evaluation (or an economic evaluation in general) is that it allows the flexibility to shift between different perspectives when considering costs to calculate the ICER. Traditionally, a healthcare payer perspective is adopted that focuses exclusively on costs borne by the payer or health insurance. However, as a rare disease may significantly decrease the productivity of patients and caregivers (for instance by impairing their professional activity (EURORDIS 2017)), a societal perspective could be more appropriate in the context of OMPs (Jönsson 2009; Annemans et al., 2017; Lakdawalla et al., 2018), as it considers the impact on areas other than the health care sector. Shifting from a healthcare payer to a societal perspective allows the inclusion of an OMPs positive impact on productivity into the value assessment, which is the case in the Netherlands (Knight and Pearson 2019), Denmark (Knight and Pearson 2019), Finland (Knight and Pearson 2019), Norway (Knight and Pearson 2019) and Sweden (Knight and Pearson 2019).

Despite the strengths listed above, we also found barriers toward the use of standard economic evaluation for OMPs. First of all, it is argued that one universally true and “constant” ICER threshold does not exist, and, in fact, is constantly changing. Moreover, most countries do not apply an explicit ICER threshold (Iskrov et al., 2017; Eichler et al., 2004). Moreover, as we have mentioned before, it is difficult for OMPs (especially those indicated for ultra-rare diseases) to adhere to standard cost-effectiveness thresholds (Schuller et al., 2015; Drummond et al., 2007). Their uncertain effectiveness, combined with the lack of transparency surrounding the price of an OMP, leaves decision-makers with little negotiation power to lower the high prices that manufacturers set for an OMP and, as such, improve their cost-effectiveness (see Box 1) (Simoens 2011).

Also, generic health outcome measures, such as the QALY, are used to express the effectiveness of an intervention. However, the methods used to value health outcome measures may underestimate an OMPs effectiveness. On the one hand, questionnaires that are too general to capture relevant rare disease symptoms will not capture all meaningful treatment effects. This is the case with the EuroQol-5D (EQ-5D), a well-known questionnaire with several limitations regarding its use for rare disease patients (Nord et al., 2009; Schuller et al., 2015; Towse and Garau 2018). For instance, the EQ-5D does not measure an increase in walking distance, despite this being a meaningful treatment outcome for a rare disease patient that is housebound (Picavet et al., 2013a; Pinxten, et al., 2012; Douglas et al., 2015). This treatment effect could be of value in the appraisal of an OMP, especially when its generic health benefit in terms of for example QALY gains is uncertain (Towse and Garau 2018; Lakdawalla et al., 2018; Douglas et al., 2015; Picavet et al., 2013b; Pinxten, et al., 2012). On the other hand, disease-specific questionnaires may not be properly translated into a generic outcome measure and, as a result, will not allow a comparison between different diseases (Nord et al., 2009; Priedane et al., 2018).

Another downside toward the use of the QALY as a measure for an OMPs effectiveness is the fact that the value of a QALY may differ according to patients’ characteristics (Harris 1987; Kappel and Sandøe 1992; Nord et al., 2009). For instance, the amount of QALYs gained from a treatment with either an OMP or non-OMP relies on a patient’s capacity to benefit from a treatment (Cleemput et al., 2011). For a rare disease patient, this capacity is generally lower (compared to a non-rare disease patient), in particular when an OMP does not cure the disease. This is the result of the disease’s severe nature and its impact on life expectancy. This means that a framework utilizing QALYs for drug appraisal could discriminate against OMPs. On a side note, it is not clear whether society values a patient’s capacity to benefit, as preference studies showed that society places a lower value on any additional treatment benefits (such as QALYs) once a minimum amount has been obtained (Schlander et al., 2016).

Underpinning the ethical principle of equity, all patients should have an equal chance at receiving treatment, regardless of the rarity of the disease (Drummond et al., 2007). This principle has been captured in EU legislation.^2^ Moreover, following the ethical principle of non-abandonment, neglecting the needs of the currently 17.8–30.3 million European rare disease patients, representing approximately 3.5–5.9% of the population (Nguengang Wakap et al., 2020), would be considered unethical (Pinxten et al., 2012). Valuing OMPs based on efficiency criteria such as cost-effectiveness alone is considered to be unfair toward rare disease patients, as this inhibits their chance for equal treatment even more given their high unmet need, compared to non-rare disease patients.

#### Modifying the ICER Threshold

Some countries, have implemented a VAF that applies a flexible ICER threshold, either for OMPs, ultra-OMPs or both (see Table 1).

One major strength of a variable ICER threshold is that it allows for less cost-effective OMPs to be reimbursed based on legitimate HTA criteria, other than those traditionally relating to efficiency, safety or economic consequences. Allowing a higher ICER threshold for OMPs would improve access for rare disease patients and thus enable equal access to treatment between rare disease and non-rare disease patients. Furthermore, the implications of including non-traditional criteria (such as disease severity) in the decision-making process become more explicit when these criteria are linked to a higher or lower ICER threshold (Juth et al., 2020).

Nevertheless, a critical downside of modifying the ICER for OMPs is that it may discriminate against non-OMPs and thus, exacerbate unequal access between both, if the approach is not based on robust evidence (Paulden et al., 2014). For instance, the Netherlands (van de Wetering et al., 2013; Stolk et al., 2004; van de Wetering et al., 2013), vary the ICER threshold according to the severity of illness. However, such estimates of severity are believed to be uncertain and heterogeneous (Versteegh et al., 2019). Furthermore, it is not clear how a class of severity (see Table 1) should relate to a specific ICER threshold (Bobinac et al., 2012). Moreover, when falling below the ICER threshold, manufacturers may maximize their gains by filling in the gap (Côté and Keating 2012). As such, a higher ICER threshold could provide an incentive for manufacturers to make unnecessarily high-risk investments (McCabe et al., 2008).

#### Attaching Weights to QALYs

Some countries add societal preferences into standard economic evaluation (Drummond 2008), by varying the weight of a QALY according to other legitimate criteria, which could be relevant for OMPs as well (see Table 1).

When an OMP-generated QALY is given a higher weight, the OMP becomes more cost-effective and will more likely fall below the ICER threshold (Wailoo et al., 2009). Much like the modified ICER threshold approach, a VAF that applies QALY weights would increase an OMPs chance for reimbursement and thus, would improve access for rare disease patients (Dear et al., 2006; Hughes et al., 2005).

The same criticism that applies for a modified ICER threshold is also applicable for a framework weighting QALYs, namely the lack of empirical base to link equity weights, such as those depicted in the example of England and Wales, to QALYs (see Table 3) (Bobinac et al., 2012). This also means that the criteria, on the basis of which QALYs are weighted, should be based on existing societal preferences (Sassi et al., 2001; Bobinac et al., 2012). If we assume that healthcare budgets are limited, other patients will bear the “opportunity cost” of an OMP being reimbursed (Paulden et al., 2014). Hence, incorporating criteria such as “severity of disease” into the appraisal process may contribute to inequality if their inclusion is not based on empirical evidence, meaning that they should reflect existing societal preferences (McCabe et al., 2006; Linley and Hughes 2013; Simoens et al., 2013).

**TABLE 3:** QALY weights applied by NICEs HST process (adapted from: NICE Interim Process and Methods of the Highly Specialised Technologies Programme Updated to reflect 2017 changes).

Additional QALYs gained (per patient, over a lifetime)	QALY weight
≤10	1
11–29	1–3 (in equal increments)
≥30	3

QALY, quality-adjusted life-year; NICE, national institute for health and care excellence; HST, highly specialized technologies; ICER, incremental cost-effectiveness ratio.

Also, decision-makers often do not know which patients bear the opportunity cost of a positive reimbursement decision, nor the characteristics that these patients present (Wailoo et al., 2009; McCabe et al., 2010). This could be due to the fact that in general, some patient groups, such as those for rare diseases, may be more vociferous (and thus more visible) than others, even though they may not always agree among themselves. This may have led to a disproportionate exposure of societal preferences toward the treatment of rare disease patients, compared to those suffering from a disease that is less visible to decision-makers. However, it is important to keep in mind that, when the value of OMP-generated QALYs increases (by weighting QALYs), the health (or QALY gain) of those who bare the opportunity cost is valued less. Nevertheless, important yet unexposed societal preferences may exist as well. By not carefully considering the needs of those who bear the costs (and hence, unexposed societal preferences toward these patients’ characteristics), rare disease patients may be unjustly favored over non-rare disease patients (McCabe et al., 2006; Wailoo et al., 2009; Linley and Hughes 2013; Simoens et al., 2013).

Lastly, although a formula exists to adjust the weight of a QALY according to disease severity, decision-makers may wish to consider multiple evaluation criteria (such as disease severity and unmet need) simultaneously in one VAF for OMPs. However, multiple preferences are not easily incorporated into a clear and practical equation (Richardson and Schlander 2019). Ultimately, by transforming societal preferences into numbers, the outcome may become too difficult to interpret by those involved in the decision-making process (Nord et al., 2009).

#### Multi-Criteria Decision Analysis (MCDA)

MCDA (see Table 1) has increasingly been advocated as a suitable VAF for OMPs (Simoens 2014; Iskrov et al., 2016; Kanters et al., 2015; Serpik and Yagudina 2014; Baran-Kooiker et al., 2018; Friedmann et al., 2018; Gilabert-Perramon et al., 2017; Trip et al., 2014; Annemans et al., 2017). In fact, several research groups have tailored MCDA frameworks for appraisal of OMP, among which EVIDEM is currently the most researched framework for OMPs (Hughes-Wilson et al., 2012; Sussex et al., 2013b; Paulden et al., 2015; Schey et al., 2017; Wagner et al., 2016; Fedyaeva et al., 2014). Nevertheless, no health authorities have yet adopted a “full” MCDA as their standard VAF for OMP appraisal, although some countries have adopted an “MCDA-like” approach (see Table 1). MCDA’s main strength lies in the fact that it allows the flexibility to in- or exclude assessment criteria (Hughes-Wilson et al., 2012; Adunlin et al., 2015; Angelis and Kanavos 2016; Friedmann et al., 2018), so that decision-makers can include all elements which are considered to be of value for OMPs as well as non-OMPs (Wagner et al., 2016). For instance, the EVIDEM framework provides a standardized MCDA approach for health technologies in general, yet it can be tailored to disease- and/or country-specific contexts if necessary (Goetghebeur et al., 2008; Tony et al., 2011; Wagner et al., 2016).

The MCDA matrix structure allows decision-makers to keep the discussion focused around the pre-defined set of key-criteria in an objective manner (Gilabert-Perramon et al., 2017; Guarga et al., 2019). Meanwhile, the VAF still leaves room to include stakeholders’ individual comments (Guarga et al., 2019), which ultimately leads to more transparency regarding the key-arguments that led to a final decision (Thokala and Duenas 2012; Radaelli et al., 2014; Adunlin et al., 2015; Angelis and Kanavos 2016; Baran-Kooiker et al., 2018; Guarga et al., 2019). For some, this structured process of identifying and appraising criteria may be even more valuable than a final, numerical score (Wagner et al., 2016; Gilabert-Perramon et al., 2017; Guarga et al., 2019). In part, this could be due to the fact that the VAF enables decision-makers to consider uncertainty in a more deliberate manner, for example by weighting criteria differently (Friedmann et al., 2018) or by allowing score ranges when assessing criteria performance (Wagner et al., 2016). For instance, the EVIDEM framework considers different sub-criteria for the criterion “quality of evidence”. Each sub-criterium is connected to a specific disease area and can be assessed differently according to the quality of data that is common in each field. Such categorization of data quality seems appropriate in the field of rare diseases, where well-powered and double-blind RCTs are often lacking (Wagner et al., 2017).

The engagement of a broad range of stakeholders such as clinicians, patients and potentially the wider public is important within the complex decision-making process regarding OMPs. Involving them in criteria selection and weighting ensures that all stakeholder’s priorities and preferences are considered (Hughes-Wilson et al., 2012; Thokala et al., 2016; Friedmann et al., 2018; Guarga et al., 2019). Their involvement also helps to interpret the evidence from a broader range of perspectives (Guarga et al., 2019). OMP appraisal often involves trading off efficiency (i.e. cost-effectiveness) with fairness (i.e. severity, unmet need). The inclusion of patients in particular may facilitate these trade-offs between competing criteria, especially in a context of high uncertainty, as they shed light on what factors are of value in OMP treatment (Picavet et al., 2013a; Douglas et al., 2015; Marsh et al., 2017; Rosenberg-Yunger et al., 2011). This might enrich discussions and potentially lead to a better understanding of the evidence. On the one hand, a multiple stakeholder approach combined with transparent reporting helps decision-makers to justify a final decision (Iskrov et al., 2017; Baltussen et al., 2018; Kolasa et al., 2018). This is important, as the OMP appraisal process may often turn political, putting high pressure on decision-makers to avoid a negative outcome (Simoens et al., 2013). On the other hand, the outcome is more easily accepted by the wider public (Youngkong et al., 2012; Iskrov et al., 2017; Schey et al., 2017; Baltussen et al., 2018). This does not only apply for a negative reimbursement decision, since a “fair” allocation of limited funds implies that also a positive decision requires a proper justification of whether the OMP is worth its (often high) price (Simoens 2011).

When applied systematically, the decision-making process will become more consistent with subsequent appraisals (Sussex et al., 2013b; Diaby et al., 2015; Mühlbacher and Kaczynski 2016; Thokala et al., 2016; Baran-Kooiker et al., 2018). This way, MCDA meets the shortcomings of other VAFs that may consider appraisal criteria implicitly. Also, when all criteria are formalized and appraised consistently, MCDA may, in time, provide insight into the attributes that are of value to healthcare payers, patients and society as a whole. After a while, a cross-country comparison of HTA appraisal criteria and final decisions may highlight country-specific preferences for OMPs and for rare diseases, insofar as these would exist (Dharssi et al., 2017).

Despite its potential, there are several barriers toward the universal use of a standardized MCDA framework. One of the reasons can be found in the lack of consistency between models that are currently proposed or implemented. For instance, conceptual MCDA frameworks are currently not consistent in terms of criteria considered, their quantification, the methods applied to elicit criteria weights (Friedmann et al., 2018) and the MCDA-score needed to issue a positive reimbursement decision (Iskrov et al., 2016; Kolasa et al., 2016). Moreover, it is argued that a final numerical MCDA-score is difficult to interpret, as there is currently no benchmark to refer to. Baran-Kooiker et al. (2018) proposes to separate the final MCDA-score into a cost- and value-score. This would facilitate the interpretation of the score across different treatments and countries, especially since cost outcomes such as cost-effectiveness are connected to specific healthcare systems (Baran-Kooiker et al., 2018).

Another issue is the risk of double counting factors of value when there is an overlap in criteria (Marsh et al., 2018). For instance, the EVIDEM framework scores several criteria that, to some extent, relate to cost-effectiveness, for instance by including “comparative effectiveness”, “comparative safety/tolerability”, “comparative patient-perceived health/patient-reported outcomes”, and “type of therapeutic benefit”. By overvaluing the effectiveness component, the weights given to these criteria become invalid (Baran-Kooiker et al., 2018).

Although one of the framework’s strengths is that it allows to manage the uncertainty surrounding the evidence base of the OMP, MCDA does not solve the well-known barriers for evidence generation, such as the patient heterogeneity in disease profiles or the difficulty in setting appropriate clinical endpoints (Picavet et al., 2013b; Friedmann et al., 2018; Guarga et al., 2019). Also, when stakeholder groups are too small (for instance in countries with a lack of broad national expertize on clinical, patient or policy level), the weighting process becomes less robust and thus, less replicable (Sussex et al., 2013b). Lack of expertize could also create barriers for stakeholders’ thorough understanding of the rare disease and the available evidence on the OMP, which is crucial for both the weighting and scoring process (Sussex et al., 2013b).

Finally, literature suggests that economic criteria are generally considered to be less important by multi-stakeholder groups involved in the weighting of MCDA-criteria for OMP-appraisal (Friedmann et al., 2018). This could be an important hurdle for decision-makers, who are often restricted by budget limits. Moreover, health authorities may be reluctant to become too transparent, which could keep them from adopting an MCDA-framework for decision-making (Sussex et al., 2013b). This was mentioned in a research paper by Sussex et al., although the authors do not mention potential reasons for this reluctance. Additionally, one panel discussion mentioned confidentiality risks as a reason not to implement MCDA (Sussex et al., 2013a).

Some countries have implemented an MCDA-like approach (see Table 1), for which we have identified several limitations from the available literature. For instance, Radu et al. report that Romania’s score card method is not applied consistently and that the final reports contain mistakes (Radu et al., 2016). In Slovakia, there seems to be a lack of transparency regarding both the appraisal process of OMPs through MCDA and the decision-making criteria that are included in the matrix. For instance, the Impact-HTA country vignettes report that there are no clear guidelines on how to perform an economic evaluation, despite its obligation. They also note the lack of a public hearing prior to submission, with the possibility to clarify or ask questions only after submission of the dossier (Nicod and Whittal 2020). Lastly, the Lombardian VTS framework has been criticized for its complexity, limited flexibility and adaptability (Cleemput et al., 2011; Radaelli et al., 2014).

#### A Separate Framework for Ultra-OMPs

Decision-making bodies in several countries (see Table 1), appraise ultra-OMPs through a VAF that is different from those applied for OMPs and non-OMPs (see Table 1).

A separate framework might meet some of the shortcomings related to the frameworks above. For instance, in Scotland, patients and caregivers are invited to shed light on benefits or disadvantages of the ultra-OMP, which are neither captured in quality of life measures nor published in the literature.

Nevertheless, by allowing a separate appraisal of ultra-OMPs vs. non-OMPs, health authorities acknowledge that there are reasons for doing so, despite a lack in consensus on whether society prefers to prioritize treatment based on disease rarity (Hughes et al., 2005; McCabe et al., 2006; Schlander et al., 2016; Soares 2012). Furthermore, in England and Wales, the VAF (named “the Highly Specialized Technologies” or HST process) has been criticized for its vague description of requirements, which have to be fulfilled in order for (ultra-)OMPs to pass through (Henderson et al., 2020). Requirements such as *the target patient group for the technology in its licensed indication is so small that treatment will usually be concentrated in very few centers in the NHS* and *the technology is likely to have a very high acquisition cost*, imply that eligibility is decided upon in an *ad hoc* manner (Richardson and Schlander 2019). This has resulted in ultra-OMPs being subjected to the same cost-effectiveness threshold as other drugs (Henderson et al., 2020). Another concern is the fact that ultra-OMPs are disregarded by the HST process when they are not exclusively indicated for ultra-rare diseases (Henderson et al., 2020). Furthermore, one of the requirements stating that *the technology has the potential for life long use*, implies that the HST process discriminates against ultra-OMPs that are potentially curative (Henderson et al., 2020). In addition, although an evaluation committee consists of a multi-stakeholder panel (National Institute for Health and Care (NICE), 2017), their preferences are not fully incorporated, as they are not involved in the weighting of evaluation criteria (Thokala 2011). Generally, it is feared that a difference in approach between decision-making bodies in England and Wales on the one hand, and Scotland on the other, will lead to unequal access to ultra-OMPs across the United Kingdom (United Kingdom) (Henderson et al., 2020). Also, timeliness seems to be a drawback, with a study in 2018 finding that in almost all cases, it took over a year to come to a final appraisal (Cockerill and Gaebler 2018). Finally, within the SMC’s PACE program, criteria are not explicitly scored or weighted (Scottish Medicines Consortium (SMC), 2012).

### Combination of Value Assessment Frameworks

First of all, many countries have VAFs that are not easily defined, as they may consist of a combination of VAFs. For instance, in Slovakia, for both OMPs and non-OMPs, the VAF links a higher ICER threshold to the outcome of an MCDA. Second, different combinations may exist depending on whether the appraisal concerns an ultra-OMP, OMP or non-OMP. We refer to the example of Slovakia, where the VAF for ultra-OMPs applies neither MCDA nor a modified ICER threshold, as it doesn’t include an economic evaluation (Malinowski et al., 2019; Nicod and Whittal 2020). Table 4 presents, per jurisdiction, an overview of the overlap between VAFs for OMPs, ultra-OMPs and non-OMPs.

**TABLE 4:** An overview of the combinations of value assessment frameworks that are applied for non-OMPs, OMPs and ultra-OMPs across geographical Europe.

Value assessment framework	No economic evaluation			Standard economic evaluation			Variable ICER threshold			Weighted QALYs			Multi-criteria decision analysis		
	Non-OMPs	OMPs	Ultra-OMPs	Non-OMPs	OMPs	Ultra-OMPs	Non-OMPs	OMPs	Ultra-OMPs	Non-OMPs	OMPs	Ultra-OMPs	Non-OMPs	OMPs	Ultra-OMPs
Austria				✓	✓	✓									
Belgium		✓	✓	✓											
Bulgaria				✓	✓	✓									
England and Wales				✓	✓							✓^a^			✓
France		✓	✓	✓											
Germany	✓	✓	✓												
Ireland				✓	✓	✓									
Latvia				✓	✓	✓									
Liechtenstein				✓	✓	✓									
Lithuania			✓	✓	✓										
Malta				✓	✓	✓									
Poland				✓	✓	✓									
Portugal				✓	✓	✓									
Romania				✓	✓^b^	✓^b^							✓	✓	✓
Scotland				✓				✓^c^	✓^a^ ^,^ ^c^						✓
Slovakia			✓				✓	✓					✓	✓	✓
Sweden							✓	✓	✓						
Netherlands	✓	✓	✓				✓	✓	✓						

^a^ In England, Wales and Scotland an extra set of non-traditional appraisal criteria applies for ultra-OMPs.

^b^ In Romania, OMPs and ultra-OMPs receive extra points in MCDA.

^c^ In Scotland, a Patient and Clinician Engagement (PACE) meeting may be organized for OMPs and ultra-OMPs, which allows patients to share their experience with the disease. For both OMPs and ultra-OMPs a higher threshold may be accepted.

ICER, incremental cost-effectiveness ratio; MCDA, multi-criteria decision analysis; OMP, orphan medicinal product; QALY, quality-adjusted life year.

## Discussion

Previous research has focused on the identification, description or legitimacy of OMP VAFs implemented by decision-making bodies in Europe (Nicod et al., 2020; Zelei et al., 2016; Bourke et al., 2018; Picavet et al., 2014b; Szegedi et al., 2018; Nicod et al., 2017; Hughes et al., 2005; Hughes et al., 2007; Wagner et al., 2016; Annemans et al., 2017; Sussex et al., 2013b; Schey et al., 2017; Towse and Garau 2018; Garrison et al., 2018; Drummond and Towse 2014; Simoens 2012; Simoens et al., 2012). Our study adds to the literature by providing an extensive and integrated overview of the strengths and weaknesses of OMP VAFs that are cited in the literature. Here we discuss the following observations that we derived from this in-depth analysis of OMP VAFs.

First, this review has shown that VAFs other than those that apply a standard economic evaluation have been developed (and implemented in some jurisdictions) with a view to account for the specific characteristics of OMPs. VAFs such as weighting QALYs or modifying the ICER threshold do not only consider cost-effectiveness, but also account for disease severity or unmet need. Each aims to increase the ICER threshold, although the methods hereto vary between both frameworks. MCDA provides a matrix that balances all criteria considered to be relevant by decision-makers and reflects a consistent and transparent way of appraisal by visualizing the key decision-making arguments and enabling a multiple stakeholder approach. Therefore, in order to allow for a comprehensive appraisal of OMPs, we advocate the implementation of a VAF that applies assessment criteria reflecting a broad definition of value that goes beyond that captured by standard economic evaluation.

Second, despite the fact that unmet need and disease severity are often-cited arguments in favor of a special appraisal of OMPs, it needs to be emphasized that these characteristics are not associated exclusively with OMPs. As a consequence, we believe that a VAF should adopt the same assessment criteria for OMPs and non-OMPs, while recognizing that some criteria are more relevant to OMPs and that OMPs may score better on some criteria than non-OMPs. For this reason, we are in favor of MCDA as such a framework meets the “equal treatment” criterion as defined in Regulation (EC) No 141/2000.

Third, this review has indicated that all VAFs struggle from a reluctance to be transparent. This could be related to the fact that decision-makers are faced with a difficult choice between “more health” or “more consumption”, also called “taboo trade-offs” (Schokkaert 2016; Luyten and Denier 2019). Confronted with these trade-offs, decision-makers may create an “escape route”, hereby banning decision-making to closed expert commissions as a way to minimize individual responsibility (Fiske and Tetlock 1997; Schokkaert 2016; Luyten and Denier 2019). When setting up any VAF, we therefore suggest to adopt the concept of “accountability for reasonableness (A4R)”, meaning that a VAF should provide 1) transparency regarding the arguments that lead toward a final decision, while allowing 2) flexibility to reflect on decisions in light of new arguments. The framework should furthermore ensure that 3) arguments are relevant, reasonable, and based on reliable data, and that 4) regulation and enforcement are in place to ensure that aforementioned requirements are being met (Daniels and Sabin 2008).

Fourth, we disagree with the notion that clinical and financial uncertainty associated with OMPs calls for a separate VAF for OMPs. This uncertainty stems from limitations in the design and collection of OMP data, which cannot be resolved by a framework assessing the value of OMPs. Instead, other approaches are required and have been proposed in the literature such as the conduct of dose-response studies (Simoens et al., 2012), the involvement of patients to establish “clinically meaningful” endpoints before the start of a trial (Picavet et al., 2013a), and the set-up of disease-specific registries (McCabe et al., 2006). In order to manage financial uncertainty, decision-makers can adopt an opportunity cost approach, such as the one applied in the Italian region of Lombardy (cfr. supra). Another way to manage clinical and financial uncertainty associated with OMPs is by means of a managed entry agreement (MEA). These (often) confidential agreements between the healthcare payer and the pharmaceutical company link a (temporary) reimbursement decision to specific conditions, either on a financial (financial-based MEAs) and/or on a clinical level (performance-based risk-sharing MEAs) (Campillo-Artero et al., 2012; Garrison et al., 2013; Morel et al., 2013; Aho et al., 2017; Bouvy et al., 2018). A financial MEA may, for example, entail a utilization or volume cap as a means to manage an uncertain budget impact and promote rational drug use. Performance based MEAs link reimbursement to the OMPs’ performance on a patient (performance linked reimbursement) or population level (coverage development agreement, CED) (Morel et al., 2013; Wenzl and Chapman 2019). However, a recent OECD report found that, to date, most performance-based MEAs have failed to decrease clinical uncertainty (Morel et al., 2013; Wenzl and Chapman 2019). This results, among other reasons, from the fact that CED protocols generally fail to address the majority of the uncertainties which were initially identified (Pouwels et al., 2019). The authors further suggest that jurisdictions implement legislation that (at least to some extent) allows them to share information on effectiveness between countries, an idea that seems particularly relevant to rare diseases.

## Conclusion

This review has provided an overview of the principal strengths and weaknesses associated with VAFs in the context of OMPs, such as those with or without a standard economic evaluation, a VAF that weighs QALYs or varies the ICER threshold, and MCDA. Although the choice for one or the other depends on the jurisdictions’ existing framework or geopolitical context, we advise against the implementation of a separate VAF for (ultra-)OMPs. We suggest that, in setting up a VAF, decision-makers align the framework with the concept of A4R, ensuring a comprehensive approach that provides the legitimacy of the trade-offs between competing efficiency and equity values, through transparency surrounding criteria and their respective weights, hereby involving multiple stakeholders. By doing so, they subject both OMPs and non-OMPs to the same standards, while giving them an equal chance for reimbursement and honoring the principles that are laid out in the OMP legislation.

## Limitations of the Review

Our review is not without limitations. First of all, publication bias may have led to under- or overreporting of either strengths or weaknesses of VAFs implemented in certain jurisdictions. Also, our literature review was limited to studies published in English. Third, as our manuscript aims to provide a broad perspective on the topic, in addition to the lack of comprehensive publications on strengths and weaknesses of VAFs for OMPs, a narrative rather than a systematic review was chosen, inducing a potential risk of selection bias.

## Footnotes

1 Regulation (EC) No 141/2000 of the European Parliament and of the Council of 16 December 1999 on orphan medicinal products.

2 Regulation (EC) No 141/2000.
